# Midfrontal neural dynamics distinguish between general control and inhibition-specific processes in the stopping of motor actions

**DOI:** 10.1038/s41598-019-49476-4

**Published:** 2019-09-10

**Authors:** Jakob Kaiser, Natalie Annette Simon, Paul Sauseng, Simone Schütz-Bosbach

**Affiliations:** Ludwig-Maximilian-University, D-80802 Munich, Germany

**Keywords:** Cognitive control, Human behaviour

## Abstract

Action inhibition, the suppression of action impulses, is crucial for goal-directed behaviour. In order to dissociate neural mechanisms specific to motor stopping from general control processes which are also relevant for other types of conflict adjustments, we compared midfrontal oscillatory activity in human volunteers via EEG between action inhibition and two other types of motor conflicts, unexpected action activation and unexpected action change. Error rates indicated that action activation was significantly easier than the other two equally demanding tasks. Midfrontal brain oscillations were significantly stronger for inhibition than for both other conflict types. This was driven by increases in the delta range (2–3 Hz), which were higher for inhibition than activation and action change. Increases in the theta range (4–7 Hz) were equally high for inhibition and change, but lower for action activation. These findings suggest that inhibition is facilitated by neural mechanisms specific to motor-stopping, with midfrontal delta being a potentially selective marker of motor inhibition.

## Introduction

When changing circumstances render prevalent actions as no longer beneficial, we need to be able to stop them. The suppression of prepotent behavioural tendencies is called inhibition^1^. Successful inhibition of action impulses is believed to play an important role in avoiding and overcoming harmful behavioural patterns, such as seen in addictions, overeating or obsessive-compulsive disorders^2,3^. Since inhibition implies the absence of a directly observable action, the investigation of this phenomenon is difficult at the behavioural level. Therefore, identifying neural underpinnings of inhibitory control could greatly benefit our understanding of how to stop unwanted action impulses.

Inhibition of intended actions has regularly been found to lead to an increase in low-frequency oscillations in the delta-theta range (<7 Hz) in medial frontal brain regions as assessed via EEG^4–6^. Based on such findings one might speculate that these midfrontal oscillations at least in part represent a neural marker of inhibition. For example, they might be related to a proposed fronto-basal network for motor suppression, involving the supplementary motor area (SMA), the inferior frontal cortex and the basal ganglia^7–10^.

However, increases in midfrontal low-frequency oscillations appear to be not exclusive to inhibition. Similar neural oscillatory patterns have been found in a wide variety of contexts in which a conflict arises that affords readjustments of ongoing behaviour, such as task switching^11^, or post-error processing^12,13^. While most studies investigated these midfrontal oscillations in the theta range (4–7 Hz), they sometimes have also been reported for the delta range (<4 Hz)^4,14,15^.

Since midfrontal low-frequency oscillations can be found in many different task contexts, it has been suggested that they are part of a general mechanism for facilitating cognitive control^16–18^. On a neural level, they might reflect activity in the cingulate cortex, which has been related to the identification and resolution of behavioural conflicts^19,20^. Cognitive control refers to neural processes aimed at facilitating cognitive and behavioral adjustments for overcoming goal-relevant conflicts. In part, the type of adjustments necessary for successfully resolving a conflict strongly depends at the task at hand. For example, while outright inhibition of an intended motor action can represent an appropriate conflict resolution in some task contexts, other types of conflicts might afford the enactment of a new motor action instead^21,22^. Despite the task-specificity of conflict adjustments, it has been suggested that different conflict tasks partially rely on a set of overlapping neural processes for detecting conflicts and initiating cognitive control^17,19^. Such processes could be described as general cognitive control mechanisms, since they are relevant in different types of tasks. Here, midfrontal low-frequency oscillations represent one possible marker of general control processes^16^. However, the exact role of midfrontal low-frequency oscillations for inhibition is not yet clearly understood. Importantly, since inhibition conflicts elicit both general control and inhibition-specific neural processes, previous studies cannot clearly distinguish between oscillatory responses which are specifically related to motor stopping as compared to neural reactions related to general control processes. For understanding the neural underpinnings of successful motor inhibition, it would be important to know in how it relies on mechanisms that are specific to motor stopping compared to general control processes which are also evoked in other conflict adjustments.

One way to induce motor conflicts is to let participants repeatedly perform a standard action, which then unexpectedly must be replaced by another type of response. Since the frequent performance of the standard action leads to the formation of a prepotent motor tendency, the sudden change towards the infrequent response behaviour affords increased cognitive control to withstand the inclination to elicit the prepotent motor action. Thus, the need to quickly divert from the standard response pattern creates a motor conflict. For example, in the classic Go-NoGo paradigm, inhibitory conflicts are typically induced by intermixing frequent (prepotent) button presses with the infrequent (conflict-inducing) withholding of button presses^23^. In order to identify inhibition-specific processes, some previous studies compared motor inhibition induced in Go-NoGo tasks with alternative types of motor conflicts (for reviews see^21,24^). For example, some previous experiments contrasted inhibition (frequent Go and infrequent NoGo trials) with motor activation conflicts, where on most trials no action had to be performed, while some trials afforded a sudden button press (frequent NoGo and infrequent Go trials)^25^. Concerning midfrontal oscillations, results of one study showed increased midfrontal theta responses during inhibition compared to activation conflicts^26^. This could indicate that midfrontal oscillations partly reflect an inhibition-specific neural process. However, inhibition conflict tasks compared to activation conflict tasks usually lead to higher error rates, which suggests differences in task difficulty and, thus, increased demands for cognitive control ressources^26,27^. Thus, it might be more difficult to quickly suppress a frequently performed action (inhibition conflict) compared to a situation in which one occasionally has to perform a button press, which is interleaved with longer periods of inaction (activation conflict). Accordingly, previous studies leave open the question in how far stronger midfrontal oscillatory responses for inhibition compared to activation conflicts reflect inhibition-specific processes as compared to differences in general, non-specific control demand due to the increased difficulty of action inhibition.

The goal of the present study was to clarify the role of midfrontal oscillations in inhibition, by comparing inhibition with different types of motor conflicts. In each case we only changed the type of frequent (prepotent) and infrequent (conflict) action while keeping all other aspects of the tasks identical. As in previous studies, we employed a task inducing inhibition conflicts (infrequent stopping after periods of frequent button presses) and contrasted it with a task inducing activation conflicts (infrequent button presses after periods of frequent inactivity). Importantly, we additionally employed a task evoking conflicts due to a sudden change in motor action (infrequent pressing of one alternative button after frequent pressing of another key). Compared to (potentially less demanding) activation conflicts, a sudden change of an action has previously been found to lead to a comparable degree of control demand as motor inhibition^28^. Using EEG, this allowed for dissociation between inhibition-specific and general control aspects of midfrontal oscillatory reactions in conflict-induced motor adjustments. Additionally, we estimated the neural sources of low-frequency oscillations in midfrontal brain areas which were sensitive to the occurrence of motor conflicts across the different types of motor conflicts (action inhibition, activation or change).

## Results

### Error rates

Mean rates of errors are shown in Table 1. Analysis of error rates indicated a significant main effect of TRIAL TYPE, F(1, 23) = 73.18, p < 0.001, ${\eta }_{p}^{2}$ = 0.76, a main effect of TASK, F(2, 46) = 33.02, p < 0.001, ${\eta }_{p}^{2}$ = 0.59, as well as TRIAL TYPE*TASK interaction, F(2, 46) = 15.77, p < 0.001, ${\eta }_{p}^{2}$ = 0.41. In all three tasks, conflict trials lead to more errors than prepotent trials: inhibition: *t*(23) = 6.80, p < 0.001, d = 1.39, activation: *t*(23) = 3.29, p = 0.003, d = 0.67, change: *t*(23) = 7.39, p < 0.001, d = 1.51. This increase in error rate from prepotent to conflict trials (i.e., difference in error percentage between conflict and prepotent trials) was significantly higher for the inhibition than the activation task, *t*(23) = 5.09, p < 0.001, d = 1.04, and significantly higher for the change than the activation task, *t*(23) = 5.54, p < 0.001, d = 1.13. In contrast, inhibition compared to change conflicts did not lead to a significant increase in errors, *t*(23) = −0.96, p > 0.90, d = 0.20. This indicates that the activation task was easier for participants, while the degree of difficulty did not significantly differ between motor inhibition and motor change.

**Table 1:** Mean error rates in percentages (with standard deviations) for all adjustment tasks.

Trial Type	Conflict Task		
	Inhibition	Activation	Change
Conflict Trials (25%)	13.0% (9.7)	4.4% (6.4)	16.3% (9.8)
Prepotent Trials (75%)	1.7% (2.7)	1.0% (1.9)	2.9% (3.9)

### EEG frequency analysis

Analysis of baseline-corrected oscillatory activation indicated a significant main effect of TRIAL TYPE, F(1, 23) = 112.20, p < 0.001, ${\eta }_{p}^{2}$ = 0.83, a significant effect of TASK, F(2, 46) = 3.85, p = 0.03, ${\eta }_{p}^{2}$ = 0.14, as well as a TRIAL TYPE*TASK interaction, F(2, 46) = 9.24, p < 0.001, ${\eta }_{p}^{2}$ = 0.29. This indicates that the conflict types differed in their midfrontal reactivity. In all tasks, midfrontal oscillatory responses were stronger for conflict compared to prepotent trials, inhibition: *t*(23) = 11.88, p < 0.001, d = 2.43, activation: *t*(23) = 3.68, p = 0.001, d = 0.75, change: *t*(23) = 4.66, p < 0.001, d = 0.95. The increase in conflict-elicited responses was significantly stronger for the inhibition than the activation task, *t*(23) = 3.89, p < 0.001, d = 0.79. Additionally, midfrontal responses were significantly stronger for inhibition than for change, *t*(23) = 3.18, p = 0.004, d = 0.65. Activation and change did not differ significantly in their conflict-induced increases, *t*(23) = −1.13, p = 0.80, d = 0.23. Thus, all three conflict types induced a midfrontal control signal, with inhibition leading to a significantly stronger midfrontal response than any other type of motor conflict.

**Figure 1:**
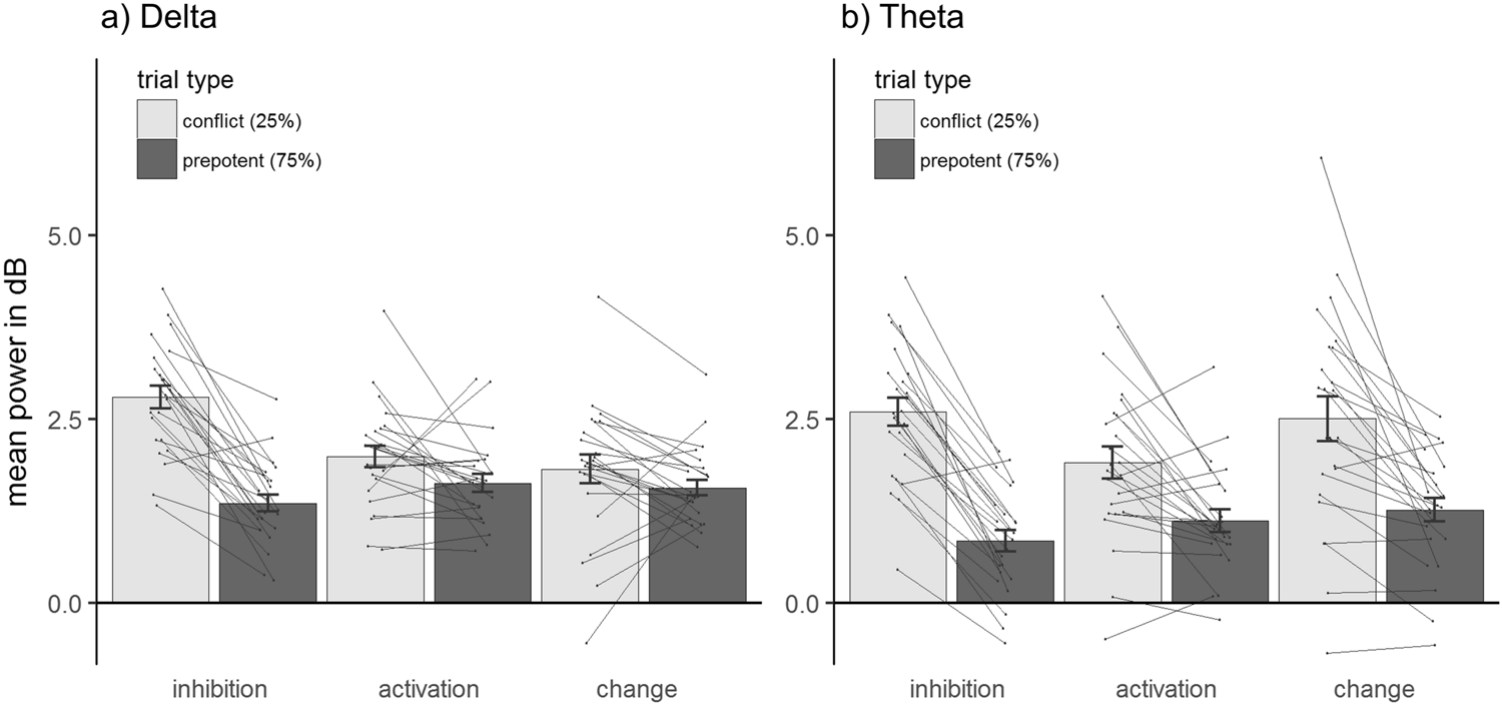
Mean power increases in dB between 0.2 to 0.6 seconds after stimulus onset separately for (**a**) delta (2–3 Hz) and (**b**) theta (4–7 Hz) frequencies for all conditions. Error bars show +−1 standard error. Dots represent individual data points with lines connecting data points of the same participant within each task.

In order to test for potential differences between the frequency bands involved, we repeated our analysis separately for the delta and theta band. Adding FREQUENCY (delta/theta) as a factor to the ANOVA led to a significant FREQUENCY*TRIAL TYPE*TASK interaction, F(2, 46) = 4.98, p = 0.01, ${\eta }_{p}^{2}$ = 0.18 (qualifying main effects of FREQUENCY, TRIAL TYPE, TASK, as well as a FREQUENCY*TRIAL TYPE and a TRIAL TYPE *TASK interaction, all p’s < 0.03, cf. Fig. 1 for condition-wise mean values). This indicates that the conflict tasks differed in their reactions in the delta and theta range. For delta, the increase in oscillatory power was significantly stronger for the inhibition than the activation task, *t*(23) = 3.81, p = 0.03, d = 0.78. Additionally, delta was significantly stronger for inhibition than for change, *t*(23) = 5.64, p < 0.001, d = 1.15. Activation and change did not differ in their delta increase, *t*(23) = 0.54, p > 0.90, d = 0.11. For theta, the conflict-related amplitude increase was significantly stronger for inhibition than for activation, *t*(23) = 3.53, p = 0.005, d = 0.72, but did neither differ significantly between inhibition and change, *t*(23) = 1.81, p = 0.25, d = 0.37, nor between change and activation, *t*(23) = 1.60, p = 0.37, d = 0.33.

**Figure 2:**
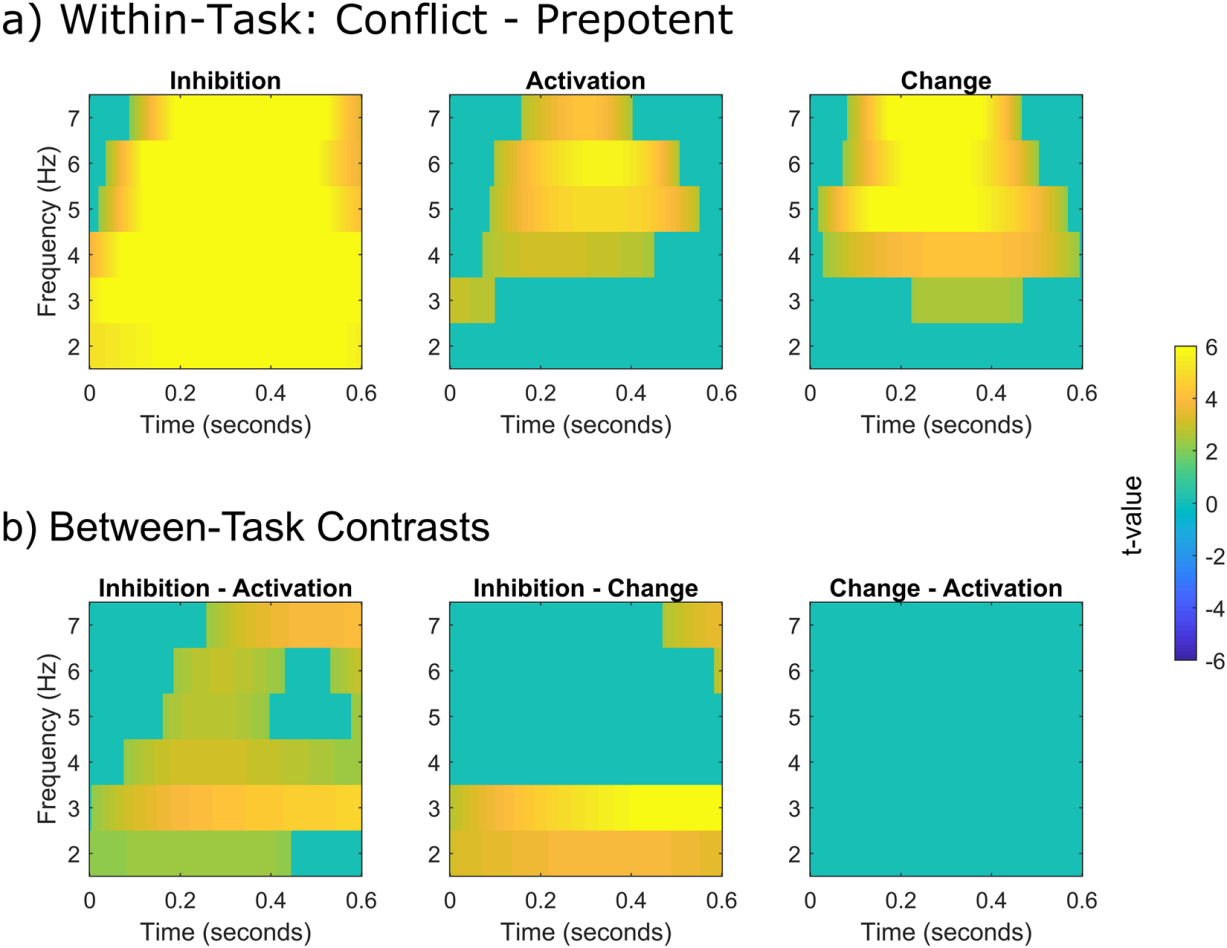
Statistical maps showing significant contrasts for each time-frequency point between averaged activity of conflict and prepotent trials within each task, as well as the contrasts in conflict induced activity (conflict − prepotent trials) between the tasks. Each statistical map is controlled for multiple comparisons via false discovery rate adjustments. Nonsignificant points have been masked as 0.

Figure 2 shows the results of the statistical analysis for individual time-frequency points. As indicated by the analysis of the averaged data, all tasks showed significant increases in activity for conflict compared to prepotent trials. Concerning the differentiation between delta and theta, it is noteworthy that the inhibition task and, to a lesser extent, the change task both showed conflict induced increases throughout the delta/theta range. On the other hand, significant increases for the activation task appeared to be mostly confined to the theta range (cf. Fig. 2a). The comparison of the conflict-induced increases between the tasks (Fig. 2b) shows significantly higher effects for inhibition compared to activation within both the delta and the theta range. For the contrast between inhibition and change, significant effects emerge mostly in the delta range. To conclude, overall midfrontal increases in oscillatory power were significantly stronger for inhibition than other types of motor conflicts. This was mainly driven by conflict-induced changes in the delta-band which were significantly higher for inhibition than for both activation and change conflicts. Theta activity was higher for inhibition than for activation but did not clearly differentiate inhibition from motor change.

### Source estimation

**Figure 3:**
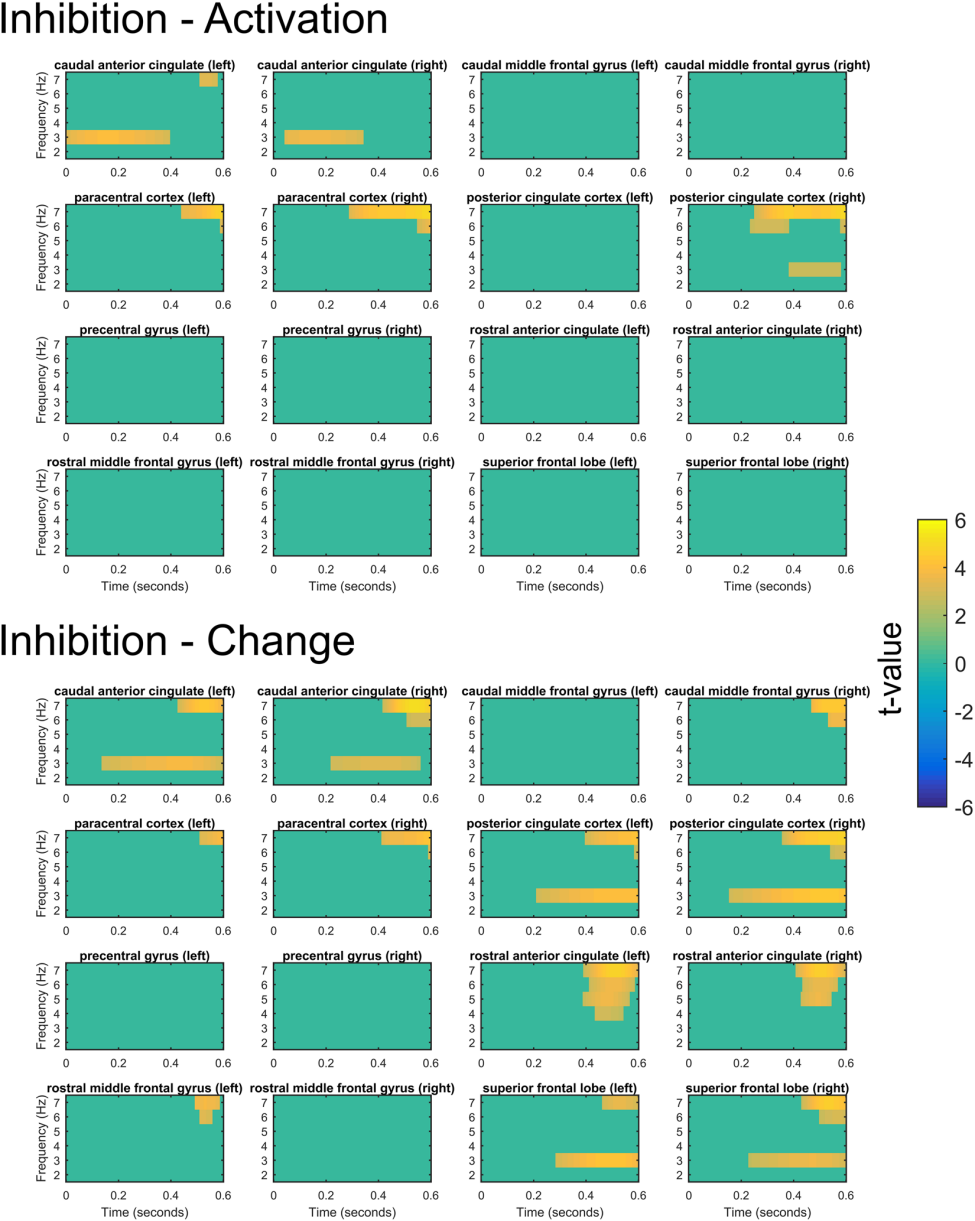
Statistical maps showing significant contrasts between tasks conflicts for each time-frequency point for source-reconstructed regions of interests (ROIs) in midfrontal to frontal regions. Each contrast compares conflict-induced activity (defined as the average difference between conflict and prepotent trials in each task) between two tasks. Statistical maps are controlled for multiple comparisons via false discovery rate adjustments. Nonsignificant points have been masked as 0. The contrast between the change and activation task is omitted, since no significant differences were found here.

Figure 3 shows the statistical maps for the comparisons of conflict-induced activation (conflict trials – prepotent trials) between inhibition and the other two tasks in ROIs of source-reconstructed data. For the contrast between the change and activation task, no significant differences emerged in any of the ROIs. For inhibition compared to the activation task, during the initial phase of the trials stronger effects for inhibition emerged at the caudal anterior cingulate cortex. Additionally, to the end of the trial, inhibition compared to activation also showed significant increases for the posterior cingulate and paracentral cortex. Note that the response time maximum throughout the experiment was 600 ms, and hence neural processes related to conflict resolution were most likely to take place in the earlier time period of the trial. Accordingly, significant differences at the end of the time period could also be related to post-response processes. For inhibition compared to motor change, significant differences emerged at the anterior caudal cingulate cortex, as well as the posterior cingulate cortex. Additionally, the rostral anterior cingulate cortex, as well as the frontal lobe showed prolonged significant differences in the later stage of the trial. Overall, comparison of inhibition with other motor tasks in source space suggests that differences in midfrontal low-frequency activity were mostly driven by increased activation of the caudal anterior cortex and the posterior cingulate cortex.

## Discussion

Previous studies found midfrontal oscillations in the delta-theta range both during motor inhibition^4,6,29^, as well as other cases of behavioural adjustments affording increased cognitive control^13,30,31^. The current study contrasted motor inhibition with two other common types of action conflicts: sudden motor activation and sudden motor change. This allowed us to distinguish between inhibition-specific and general control-related reactivity in midfrontal oscillations. For each motor task the conflict response led to increased midfrontal low-frequency activity. Importantly, inhibition compared to other motor conflicts led to significantly stronger midfrontal reactions. Midfrontal low-frequency oscillations have repeatedly been observed during conflict tasks and are commonly interpreted as part of a general (i.e. not task-specific) mechanism for enacting cognitive and behavioural control^17,20^. Thus, the finding that inhibition selectively elicited a stronger midfrontal response is of great relevance for our understanding of this frequently reported oscillatory response, as it refines our understanding of the role of midfrontal activity for inhibition and motor control. Importantly, in the current study the motor inhibition task and the motor change task showed a comparable rate of errors and obviously were equally demanding. This strongly suggests a specific midfrontal involvement in inhibition rather than differences due to mere task difficulty.

While midfrontal control signals are often reflected in both the delta and theta range, previous studies of midfrontal control signals mostly focused on theta reactivity^4^. In the current study, inspection of the time-frequency data suggests that differences between inhibition and the other tasks were more likely to occur in the delta-range rather than in the theta-range. Some previous experiments suggested that delta and theta might contribute separately to motor inhibition^32,33^. For example, it has been found that delta activity but not theta activity increased during inhibition of more frequently repeated (i.e. potentially more prepotent) actions^32^. However, these studies did not compare inhibition with other types of behavioural conflicts, and therefore cannot delineate between the contributions of inhibition-specific compared to more general control processes for such findings. The results of the present study suggest that midfrontal delta activity is a more selective marker of motor inhibition, while theta activity does not allow for a clear distinction between inhibition and motor conflicts with a similar degree of control demands.

It should be noted, however, that the current findings do not suggest that midfrontal oscillatory activity in the delta and theta range represent independent processes. In fact, despite the differences in delta and theta reactivity, inspection of the time-frequency maps shows a considerable overlap in their response. Increases in the delta and theta band occurred simultaneously, suggesting they are both part of one complex oscillatory signal. It must also be pointed out that the results of the time-frequency analysis inevitably represent some degree of imprecision concerning the activity in each individual frequency band. That is, estimations of the power in one frequency band are influenced by the activity in adjacent bands^34^. Therefore, suggestions concerning a potential dissociation between the adjacent delta and theta band should be considered with caution. Nevertheless, our results indicate, that a differentiation between lower frequency bands (~delta) and higher frequencies (~theta) within the midfrontal control signal could lead to new insights concerning the neural correlates of inhibition and other types of control conflicts.

In discussing the relevance of oscillatory measures for the study of inhibition and conflict resolution, it is worth noting that these processes have also been linked to event-related potentials (ERPs) in midfrontal regions. More specifically, conflict inhibition is known to elicit the N2/P3-complex^25,35,36^. Given the overlap in their spatial and temporal occurrence, it is reasonable to assume that N2/P3 and midfrontal delta/theta at least in part an expression of the same underlying, neural mechanisms^32^. One approach to disentangle oscillatory activity from event-related potentials is the calculation of non-phase-locked oscillatory activity, which is assumed to lower the impact of ERPs on time-frequency results. The analysis of non-phase-locked time-frequency data in the current study showed clear evidence for midfrontal effects in the delta/theta range. This is in line with previous research showing that the removal of phase-locked activity (i.e., event-related potentials) did not significantly diminish conflict-induced effects on midfrontal oscillatory activity^37–39^. Overall, these findings would support the view that midfrontal oscillations are not solely an expression of event-related potentials such as the N2/P3-complex. However, it should be noted that currently established analysis techniques do not allow for an unambiguous disentanglement of ERPs and oscillatory activity^40,41^. The exact relation between event-related activity and oscillations as indicators of the same cognitive phenomena is rather complex and not yet clearly understood^42,43^. Therefore, further research is needed to clarify the relation and potential dissociation of conflict-related ERPs and oscillatory activity related to the midfrontal control signal.

Our current results do not imply that low-frequency activity during conflict resolution is confined to the midfrontal brain areas. For example, some previous studies have observed increased low-frequency activity in parietal areas during behavioural conflict adjustments, which might be an indicator of frontoparietal network related to attentional and cognitive control^11,18^. We focussed on midfrontal delta/theta activity, since previous research suggests it as an essential hub for the detection and resolution of behavioural conflicts^20^. Source estimation of the current data suggests that the difference between inhibition and other motor conflict tasks was most strongly pronounced in anterior and posterior cingulate cortex. Previous studies identified the anterior cingulate cortex as a main source for neural activity related to conflict resolution in range of different types of conflict tasks^9,41,44^. Taken together with the current findings, this might suggest that inhibition and other conflict tasks share overlapping neural sources for the generation of the midfrontal control signal, but that inhibition compared to other motor conflicts leads to a stronger response in the low-frequency range. It should be noted that the precision of source estimation in the current study is necessarily limited by the fact, that we did not have access to individual brain topographies, but instead relied on a standardized brain template. Importantly, since our analysis was focused on the source of midfrontal low-frequency oscillations our findings do not rule out the existence of inhibition-specific networks which might be crucial for inhibition but are not necessarily reflected in frontal delta/theta oscillations.

A precise dissociation between neural mechanisms specific to inhibition and other potentially related control functions remains a persistent problem for neuroscientists. There are two related reasons for this: First, successful inhibition relies on several cognitive functions generally necessary for behavioural readjustments such as detection of stimulus deviations (such as the No Go-signal in a stream of Go-signals) and motor planning^21,24,45^. Secondly, cognitive control tasks which do not necessitate an outright motor stopping in conflict situations nevertheless can evoke inhibitory tendencies. For example, surprising events as well as error-related feedback are known to evoke motor slowing, which is believed to rely on a partial activation of motor inhibition networks^5,46,47^. In these cases, the partial enactment of motor stopping tendencies could help to avoid automatic and potential erroneous responses and thus might facilitate goal-directed readjustment of behavioural tendencies, for example in the form of a speed-accuracy trade off^48,49^. Thus, it might be possible to characterize different cognitive control tasks with respect to their degree of inhibitory involvement. Based on the present findings, one could speculate that control conflicts that evoke a stronger tendency to withhold or delay motoric reactions (e.g., in order re-evaluate the next response) show stronger increases in the delta range than cognitive conflicts where the next motor response is less uncertain, even when these tasks are matched for their overall demand on cognitive control resources. However, future research is needed to test those assumptions, for example by comparing control conflicts that differ in their degree of motor involvement^13^.

To conclude, previous studies observed midfrontal low-frequency oscillations both in motor inhibition and other tasks demanding behavioural adjustments due to action conflicts. The present study presents evidence suggesting that the midfrontal oscillatory response is selectively higher for inhibitory conflicts. This effect appears to be due to a differential response in the delta and theta band: Theta activity did not differentiate between inhibition and the equally demanding motor change task. This could indicate that theta waves more strongly reflect control demands that are independent of the specific type of required motor adjustment, whereas delta waves, however, are selectively increased for outright motor stopping.

## Method

### Participants

Participants were 27 right-handed students voluntarily taking part for either course token or financial reimbursement. Three participants were excluded from statistical analysis due to too small numbers of artefact-free EEG trials (see below). The resulting sample consisted of 24 participants (16 female) with a mean age of 25.79 years (SD = 4.55). All participants provided written informed consent before the experiment. The study was approved by the local ethical board at the Department of Psychology of the Ludwig-Maximilian-University Munich, and the procedure was in accordance with its guidelines.

### Apparatus and measurement setup

The experiment was presented on a 24-inch monitor at a distance of approximately 90 cm from the participants. In this study we used the three stimuli square/circle/triangle as response cues, each of which indicated one of three possible actions: Go (pressing a button), No-Go (not pressing any button), Switch-Go (pressing an alternative button). The assignment between symbols and actions was randomized for each participant and remained constant throughout the experiment. Each of the three stimuli was presented in white on a grey background in the centre of the screen with a visual angle of 0.8°.

EEG was recorded with a BrainVision QuickAmp amplifier using 65 active electrodes (Brain Products ActiCap), positioned according to the international 10–20 system. The FCz was used as online reference, and an additional ground electrode was mounted on the AFz-position. Impedances were kept below 15 kOhm. EEG was recorded with 500 Hz sample rate and an online filter with a bandpass between 0.016 Hz and 250 Hz.

### Procedure

The experiment was divided in three tasks (inhibition/activation/change), each presented in separate blocks. The order of the tasks was counterbalanced between participants. Each task induced a motor conflict by contrasting one type of frequent/prepotent action, presented in 450 (75%) of all trials, with one type of infrequent/conflict action, presented in 150 (25%) of the trials, resulting in 600 trials per task. The order of prepotent and conflict trials was randomized separately for each individual task. The to-be-performed responses differed across tasks as follows: In the *inhibition task*, participants had to press the down-arrow button in prepotent trials (75% Go), but to not press any button in the conflict trials (25% No-Go). In the *activation task*, participants had not to press any button in the prepotent trials (75% No-Go), but to press the down-arrow button in the conflict trials (25% Go). In the *change task*, participants had to press the down-arrow button in the prepotent trials (75% Go), but to press the up-arrow button instead in the conflict trials (25% Change-Go). At the beginning of each task, participants were informed about the relative frequency of Go, No-Go or Change-Go trials in the upcoming sequence of trials, respectively. Participants performed a short training block consisting of 16 trials prior to each task, which was repeated in cases where instructions were obviously not followed correctly. Participants were instructed to respond by using their right index finger throughout the whole experiment. The timing of events was kept constant across all three tasks: Each trial started with the presentation of one response cue for 100 ms, which indicated the action the participant had to perform. Participants were instructed to respond as quickly and accurately as possible within a 600 ms time window starting with the onset of the cue. An error message was shown if participants incorrectly pressed a button in No-Go trials, pressed the wrong or no button in Go/Change-Go trials or gave the response too late. If participants responded correctly, no feedback was provided. Each trial was separated by a blank screen for a jittered duration of 1500 to 2000 ms.

### Data preprocessing

The data was filtered (low-pass: 30 Hz) and referenced to an average of all electrodes offline. For six participants one noisy electrode, and for one participant two noisy electrodes were replaced by spherical interpolations in EEGLAB^50^. Data was segmented into epochs from −1500 ms to +1500 ms relative to response cue onset. Components representing eye blinks were identified and removed using independent component analysis in EEGLAB, leading to a removal of 1–6 (mean = 1.6) components per participant. Trials with maximum deflections of +−80 µV were deleted to account for noise artefacts, resulting in the removal of, on average, 5.27% (SD = 4.58%) of all trials. Since the goal of the study was to investigate the implementation of successful conflict resolution, trials with incorrect responses were excluded from further analysis (mean rate = 4.22%, SD = 3.56%). On average, the number of remaining trials per participant and condition were 416.0 (SD = 29.0) for the prepotent actions and 124.9 (SD = 15.3) for the conflict actions.

### Frequency analysis

Time-frequency power does not only capture purely oscillatory (non-phase-locked) activity but is also influenced by event-related (phase-locked) potentials^34^. This raises a challenge for a distinction between event-related potentials and oscillatory phenomena within the same time-range. Since the focus of our investigation was the role of midfrontal oscillations, we subtracted phased-locked activity from the overall neural activation prior to time-frequency calculation. More specifically, for each condition we calculated the average event-related potential by taking the mean of all trials. The condition-wise event-related potential was then subtracted from each single trial of that condition. This approach is commonly used to minimize the influence of event-related activity on time-frequency measures and, thus, provide a purer measure of non-phase locked, i.e. oscillatory activity^37,51,52^ (but see Discussion for a further consideration of this issue). Time-frequency power was calculated around cue onset, based on the extracted 3-second epochs to avoid edge artefacts. Frequency calculation was performed in Brainstorm using Morlet Wavelets from 2–20 Hz in 1-Hetz steps^53^. Basis for the wavelet generation was a template waveform (so-called ‘mother wavelet’) with a central frequency of 1 Hz and a temporal resolution of 2 seconds FWHW (full-width at half maximum). For each individual frequency bands, target wavelets were generated by scaling the template waveform accordingly. The resulting time-frequency data was averaged for each condition. The power values at each time point were baseline-corrected via a decibel conversion relative to the frequency-specific average power between 300 and 100 ms prior to stimulus onset (dB values = 10 *log10[power/baseline]). The length of the baseline time window is similar to other studies concerning low-frequency oscillations^4,28,54^. Since in wavelet analysis each individual data point of the time-frequency results is estimated via weighted averages of surrounding time points, choosing a relatively short baseline is appropriate for time-frequency data^34^.

### Statistical analysis

In order to assess participants’ performance, the mean rate of error trials from each condition was submitted to a 3*2 ANOVA with the within factors TASK (inhibition/activation/change) and TRIAL TYPE (prepotent trial/conflict trial).

**Figure 4:**
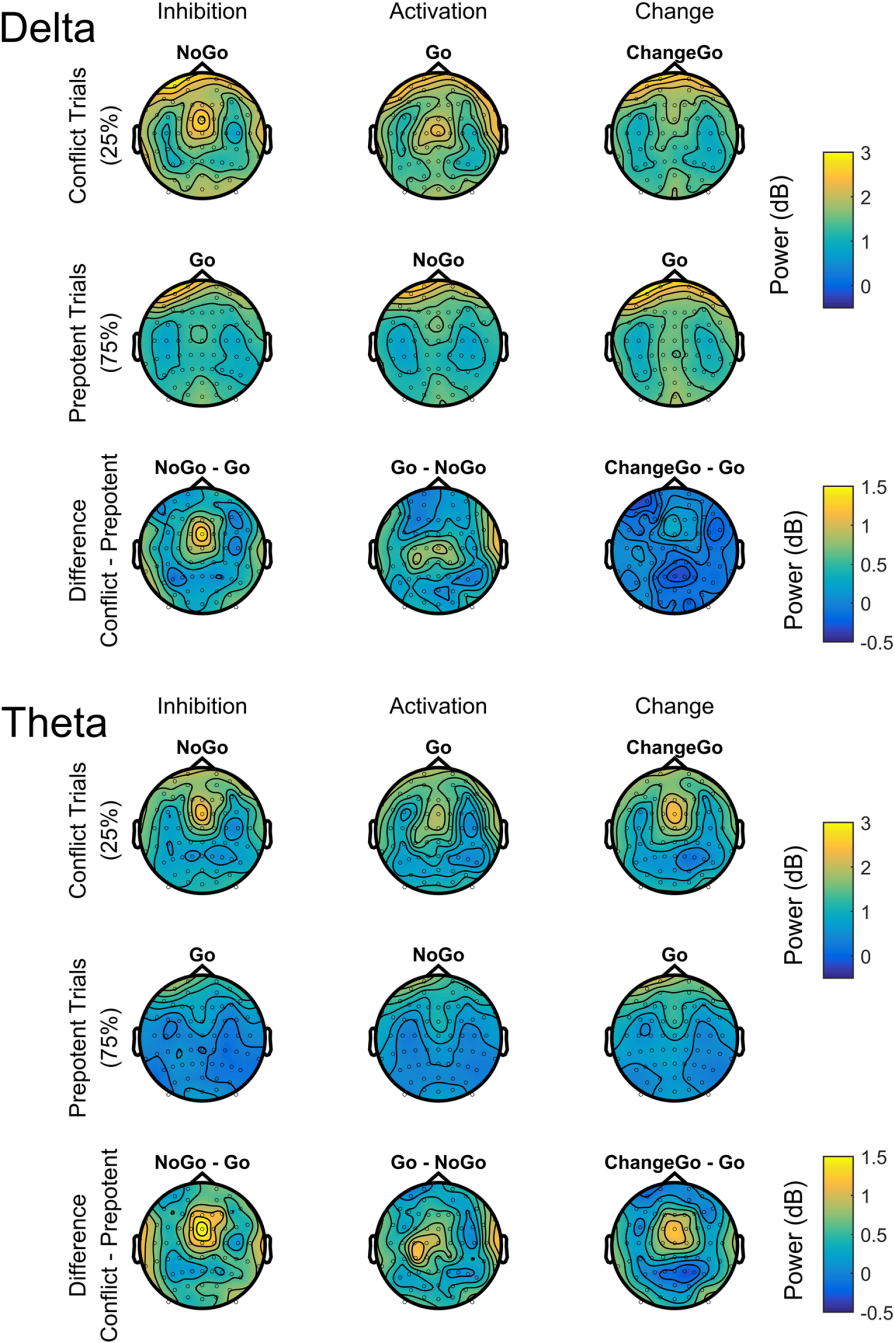
Topographical plots representing oscillatory power between 0.2 to 0.6 seconds after stimulus onset separately for (**a**) delta (2–3 Hz) and (**b**) theta (4–7 Hz) frequencies.

**Figure 5:**
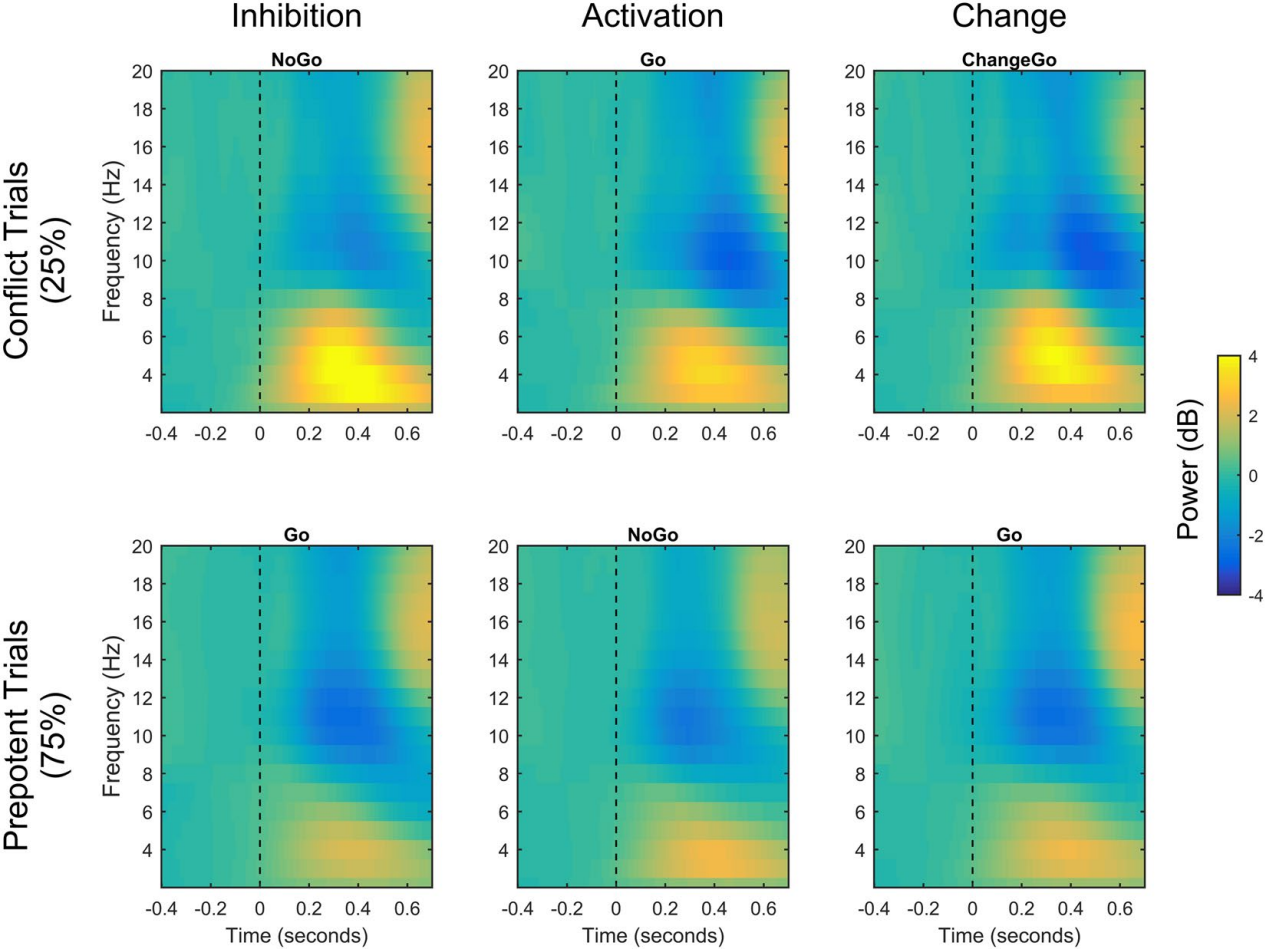
Time-frequency plots for the FCz separately for all conditions. Dashed line at time point 0 indicates action stimulus onset.

Based on previous studies of midfrontal theta, we analysed oscillatory power at the FCz electrode^4,13,55^. As can be seen in Fig. 4, this location shows a strong peak for activity in both the delta and theta frequency band in the midfrontal brain area. The time course of activity at this electrode indicated an increase of low-frequency oscillations approximately 200 ms after signal onset (cf. Fig. 5). Accordingly, we extracted activity in the delta/theta range (2–7 Hz) between 200 and 600 ms for each condition. EEG data was analysed with a 3*2 ANOVA with the two within-subject factors TASK (inhibition/activation/change) and TRIAL TYPE (prepotent trial/conflict trial). As a follow-up to the TASK*TRIAL TYPE interaction, the difference in conflict-induced reactions between the three tasks was investigated by calculating the relative increase in oscillatory power (difference between conflict and prepotent trials) for each task and comparing these difference scores between tasks. In a subsequent step, we also repeated our analysis separately for theta (4–7 Hz), as well as delta oscillations (2–3 Hz). This allowed testing for potential differentiations between these frequency bands, as well as facilitated comparability with previous studies of midfrontal control oscillations which often exclusively focused on the theta range^4,6^. Greenhouse Geiser corrections were applied for ANOVAs in case of violations of sphericity, with corrected p-values and original degrees of freedom being reported. Post-hoc comparisons were Bonferroni-corrected. All statistical tests were calculated in R using the packages *ezANOVA* and *effsize*.

Since the analysis of summary scores based on averaged time-frequency points might obscure information about the extent of individual effects, we also calculated statistical differences between the conditions based on individual data points. More specifically, for each condition and participant, we extracted one time-frequency map of low-frequency activity during the trial period (2–7 Hz, 0–600 ms). These maps were used to calculate statistical contrasts between the individual data points of average oscillatory activity in conflict and prepotent trials in each task. For this purpose, we employed nonparametric permutation tests as implemented in the Brainstorm function *process_test_permutation2p* (using 1000 randomizations). Additionally, for each task and participant we calculated the difference map between the average activity in the conflict and prepotent trials as a measure of conflict-induced increases in oscillatory power. We then employed the same nonparametric permutation procedure to calculate the contrasts of conflict-induced increases between the three tasks (i.e., inhibition – activation; inhibition – change; change – activation). In each case, multiple comparisons were controlled for via false discovery rate correction as implemented in Brainstorm.

### Source estimation

For estimating the sources of conflict-related increases we employed source reconstruction in Brainstorm^53^. Based on the MRI template ICBM152 with 15,002 dipoles, a symmetric boundary element model (BEM) of the cortex surface with a grid resolution of 5 mm was generated as forward model. For each participant a noise covariance matrix was calculated based on the −0.3 to −0.1 pre stimulus baseline. Source reconstruction was performed with the minimum norm approach with unconstrained dipole orientation based on Brainstorm’s default parameter settings^56^. Since the focus of our study was midfrontal oscillations, we extracted from the source-reconstructed data regions of interest (ROIs) in the central to frontal region as defined in the neuroanatomical atlas Mindboggle^57^. This led to the extraction of 16 ROIs (i.e., 8 analogous regions on the left and right hemisphere): caudal anterior cingulate cortex, rostral anterior cingulate cortex, posterior cingulate cortex, superior frontal lobe, caudal middle frontal gyrus, rostral middle frontal gyrus, precentral gyrus, and paracentral cortex (cf^57^ for a detailed description of the localization procedure). For each ROI and each condition, frequency-maps were calculated for each participant for the main trial period (0–0.6 s) in the delta-theta range (2–7 Hz). Frequency maps were converted to decibel relative to the pre-stimulus baseline. The goal of this analysis was to identify potential differences in source activation between inhibition and other types of motor conflicts. Therefore, we estimated the increase in oscillatory activity in the delta-theta range due to the occurrence of a conflict within each of the three tasks and compared conflict-related increases between the tasks. More specifically, for each task we subtracted the mean activity during prepotent trials from the mean activity during conflict trials. For each ROI separately, we then contrasted this measure of conflict-induced activation between the tasks with nonparametric permutation tests across the time-frequency maps (inhibition – activation, inhibition – change, change – activation), employing false discovery rate corrections for multiple comparisons. Thus, the resulting maps indicate conflict-induced source activity which differs between the tasks.

## Data Availability

Data and materials of this study are achieved online at https://osf.io/zdtgy/?view_only=66dc9498c4d247d4b2a28e6ac9c57cb4.
